# Regulation of Reactive Oxygen Species Promotes Growth and Carotenoid Production Under Autotrophic Conditions in *Rhodobacter sphaeroides*

**DOI:** 10.3389/fmicb.2022.847757

**Published:** 2022-02-28

**Authors:** Yu Rim Lee, Won-Heong Lee, Soo Youn Lee, Jiye Lee, Min-Sik Kim, Myounghoon Moon, Gwon Woo Park, Hui Su Kim, Jeong-Il Kim, Jin-Suk Lee, Sangmin Lee

**Affiliations:** ^1^Gwangju Bio/Energy R&D Center, Korea Institute of Energy Research, Gwangju, South Korea; ^2^Interdisciplinary Program of Agriculture and Life Sciences, Chonnam National University, Gwangju, South Korea; ^3^Department of Integrative Food, Bioscience and Biotechnology, Chonnam National University, Gwangju, South Korea; ^4^Energy Resources Upcycling Research Laboratory, Korea Institute of Energy Research, Daejeon, South Korea; ^5^Department of Advanced Chemicals and Engineering, Chonnam National University, Gwangju, South Korea

**Keywords:** reactive oxygen species, cell growth, carotenoid, autotrophic conditions, *Rhodobacter sphaeroides*

## Abstract

Industrial demand for capture and utilization using microorganisms to reduce CO_2_, a major cause of global warming, is significantly increasing. *Rhodobacter sphaeroides* is a suitable strain for the process of converting CO_2_ into high-value materials because it can accept CO_2_ and has various metabolic pathways. However, it has been mainly studied for heterotrophic growth that uses sugars and organic acids as carbon sources, not autotrophic growth. Here, we report that the regulation of reactive oxygen species is critical for growth when using CO_2_ as a sole carbon source in *R. sphaeroides*. In general, the growth rate is much slower under autotrophic conditions compared to heterotrophic conditions. To improve this, we performed random mutagenesis using *N*-methyl-*N*’-nitro-*N*-nitrosoguanidine (NTG). As a result, we selected the YR-1 strain with a maximum specific growth rate (μ) 1.44 day^–1^ in the early growth phase, which has a 110% faster growth rate compared to the wild-type. Based on the transcriptome analysis, it was confirmed that the growth was more sensitive to reactive oxygen species under autotrophic conditions. In the YR-1 mutant, the endogenous contents of H_2_O_2_ levels and oxidative damage were reduced by 33.3 and 42.7% in the cells, respectively. Furthermore, we measured that concentrations of carotenoids, which are important antioxidants. The total carotenoid is produced 9.63 g/L in the YR-1 mutant, suggesting that the production is 1.7-fold higher than wild-type. Taken together, our observations indicate that controlling ROS promotes cell growth and carotenoid production under autotrophic conditions.

## Introduction

The biological fixation of CO_2_ is environmentally friendly and has the technical advantage that the process is carried out at ambient conditions. Among the biological CO_2_-utilizing technologies, CO_2_ fixation through photosynthesis of microalgae has been the most extensively studied. Microalgae and cyanobacteria have developed a metabolic pathway that efficiently fixes CO_2_ by RUBISCO (ribulose-1,5-bisphosphate carboxylase/oxygenase), which catalyzes the Calvin cycle. They have been used to produce biofuels such as biodiesel due to high oil contents in cells as a storage form of fixed CO_2_ (Abomohra et al., 2020). The production of biochemicals, such as terpenoids, bioethylene, and polyhydroxybutyrate (PHB), in microalgae has also been reported (Chaogang et al., 2010; Zhu et al., 2015; Lin and Pakrasi, 2019).

One of the chemolitotrophs, *Rhodobacter sphaeroides*, is attracting attention as an industrial cell factory producing biochemicals. It has a variety of useful metabolic pathways and can uptake CO_2_ as well as sugar and organic acids *via* the Calvin-Benson-Bassham (CBB) pathway (Orsi et al., 2021). In order to increase the productivity of PHB and biohydrogen, genetic modification, and optimization of environmental conditions including temperature, carbon source, and carbon/nitrogen ratio has been conducted (Ghimire et al., 2015; Lee Y. R. et al., 2020). However, compared to heterotrophic cultures, relatively little research on biochemical production under autotrophic conditions using CO_2_ as a carbon source has been performed.

Autotrophic cultivation is very attractive in that it can directly capture CO_2_ and convert it into high-value materials that are chemically difficult to produce. However, in most cases, the slow growth in autotrophic conditions compared to heterotrophic conditions is a major technical challenge for industrialization. For instance, the relevant heterotrophic model organism *Escherichia coli* was changed to full autotrophy *via* non-native Calvin cycle gene operation by Gleizer et al. (2019). Although it can survive in autotrophic conditions, its growth rate is much lower compared with sugar fermentation. In *Alcaligenes eutrophus*, the specific growth rate is also much higher under heterotrophic conditions than under autotrophic conditions using CO_2_ and H_2_ (Friedrich et al., 1981). To overcome this, biomass and lipid productivity of microalgae were increased through a mixotrophic culture with addition of an organic source (Liang et al., 2009).

Reactive oxygen species (ROS) are generated as a result of their cellular metabolism. They mediate diverse intracellular responses, such as growth, defense, and signaling, but critical oxidative damage also occurs in cells when their endogenous levels are increased (D’Autréaux and Toledano, 2007; de Castro et al., 2013). In *Saccharomyces cerevisiae*, ROS levels were decreased through overexpression of a cell wall integrity associated gene, resulting in enhanced ethanol tolerance and increased cell viability (Zhao et al., 2017). ROS accumulation is also highly related with lipid synthesis in oleaginous microorganisms (Zhang et al., 2019). The growing body of metabolic and physiological studies support that regulation of ROS is very important to increase cell viability and metabolite production (Dong et al., 2015; Shi et al., 2017).

Autotrophic microorganisms uptake CO_2_ to produce CO, CH_4_, and acetic acid, as well as long chain chemicals such as carotenoids. High CO_2_ concentrations cause cellular stress, but also stimulate the production of fatty acids and carotenoids in *Parachlorella kessleri* (Jesus et al., 2021). Temperature, light intensity, and gas compositions, including CO_2_ and O_2_, are crucial factors in photoautotrophic culture, and in particular light intensity greatly promotes carotenoid production (Ota et al., 2009). The production of carotenoids in abundance indicates high pigment accumulation (Qu et al., 2021). Carotenoids also protect cells from oxidative stress, because they possess antioxidant activity (Glaeser and Klug, 2005). Carotenoids are thus one of the important metabolites in autotrophic growth.

In this work, we generated YR-1 mutant with improved growth under autotrophic conditions through nitrosoguanidine (NTG)-induced mutations. The endogenous levels of H_2_O_2_ and oxidative damage was relatively lower in YR-1 mutant, resulting in higher cell viability. Notably, PHB was not accumulated, but carotenoids, including spheroidenone, hydroxyneurosporene, and neurosporene, were more produced in the mutant. On the basis of these findings, we propose that ROS regulation plays an important role in autotrophic growth and carotenoid production in *R. sphaeroides*.

## Materials and Methods

### Bacterial Strain and Growth Conditions

The *R. sphaeroides* KCTC1434 strain and YR-1 mutant were grown in Sistrom’s minimal medium without succinic acid (Sistrom, 1962). For anaerobic cultures, the precultured cells were added to 20 mL of modified Sistrom’s medium in serum bottles after being diluted to OD660 of 0.1. The cultures were incubated under light-anaerobic conditions at 30°C, 150 rpm, and purged with a gas composition of CO_2_ 10%, H_2_ 60%, and argon 30%. Cell growth was observed by measuring optical density (OD) using a spectrophotometer (BioSpectrometer, Eppendorf, Hamburg, Germany) at 660 nm.

### Mutant Selection

The mutagen treatment was performed by the modified Tanaka et al. (1991). The exponential phase cells were harvested and washed twice with tris-maleate buffer (50 mM, pH 6). The cells were treated with 50 mL of 0.4 mg/mL *N*-methyl-*N*’-nitro-*N*-nitrosoguanidine (NTG) dissolved in tris-maleate buffer for 1 h at 30°C. The suspension was sufficiently cooled in ice and sequentially washed with tris-maleate buffer and growth medium. The cell pellets were resuspended with 2 mL of growth medium and transferred to a new medium. The dominant mutants were selected by serial transfer under autotrophic conditions with 10% of CO_2_ and 60% of H_2_. The serial transfer was carried out three times, and then the appropriately diluted culture solution was spread on an agar plate to isolate a single mutant colony. To screen mutants, we randomly selected several mutants from the mutant library, and estimated growth and CO_2_ consumption. To investigate mutations in YR-1 mutant, the complete genome resequencing was carried out by Macrogen (Seoul, South Korea). Mutations were identified by comparing the sequences with the corresponding wild-type genome sequence.

### Ubiquinone Extraction and Analysis

The analysis of ubiquinone was performed by simply modified protocol (Lu et al., 2013). The harvested cells were normalized to the optical density. After washing, the cells resuspended in 200 μL of 0.01 M HCl. To destruct the cells, the suspension was incubated at 75°C for 15 min. The pellets were harvested with a centrifuge and conducted vigorously vortex with 5 mL of extraction solution (ethyl acetate/ethanol = 5:3, v/v), after that the mixture was incubated for 15 min at room temperature. Cell debris was removed by centrifugation and the filtered supernatants was analyzed using high performance liquid chromatography (1260 Infinity II, Agilent, CA, United States) equipped with an Eclipse XDB-C18 column (5 μm × 4.6 mm × 150 mm, Agilent, CA, United States). The separation was achieved isocratically using the mobile phase of methanol/isopropyl alcohol (3:1, v/v). The flow rate was 1 mL/min and the column temperature was 40°C. The injection volume was 25 μL and UV detector set up at 275 nm. The standard curve was prepared using Coenzyme Q_10_ (Sigma-Aldrich, MO, United States).

### Polyhydroxybutyrate Extraction and Analysis

The extraction of PHB was performed as described previously (Lee Y. R. et al., 2020). Briefly, lyophilized cells were reacted with a 2 mL methanol and sulfuric acid solution (85:15, v/v) containing 250 mg/L benzoic acid as an internal standard. The mixture was mixed with 2 mL of chloroform and incubated for 3.5 h at 100°C. The tubes were cooled down at room temperature, and then 1 mL of NaCl was added to each tube. The mixture was vortexed vigorously for 1 min and centrifuged at 4200 rpm for 10 min. The filtered organic phase was analyzed by gas chromatography (7890, Agilent, CA, United States) with a HP-5 capillary column (30 m, 0.25 mm ID) and a Flame Ionization Detector (FID). The injection port and detector temperatures were 180°C and 200°C, respectively. The flow rate of the helium carrier gas was 1 mL/min. The PHB polymer (363502, Sigma-Aldrich, MO, United States) was dissolved in chloroform and used to prepare a standard curve.

### Extraction and Quantification of Carotenoids

The total carotenoid extraction was carried out as described previously (Lee Y. R. et al., 2020). Briefly, 33.3 mg of dried cells was suspended in 1 mL of 3 M HCl and then incubated for 30 min at 30°C, 100 rpm. The suspensions were centrifuged for 20 min and the supernatants were discarded. The pellets were resuspended in 1 mL of acetone and incubated for 30 min. The supernatants were harvested with centrifugation and the absorbance was measured at 480 nm with sufficient dilution. The spheroidenone, hydroxyneurosporene, and neurosporene were extracted from the cell pellet using a 7:2 acetone:methanol solution and hexane, respectively. The quantification was conducted with the reported extinction coefficients (Chi et al., 2015). The millimolar extinction coefficients (1 cm path length) used were 122 for spheroidenone at 482 nm in the acetone:methanol mixture, 149.4 for hydroxyneurosporene at 438 nm in hexane, and 159.4 for neurosporene at 438 nm in hexane.

### Transcriptome Analysis

Total RNA for RNA sequencing was isolated by using a Quick-RNA Fungal/Bacterial Miniprep kit (Zymo Research, Irvine, CA, United States). RNase-free DNaseI was treated to total RNA to eliminate any contaminating genomic DNA. Complementary DNA library construction and raw data processing for transcriptome analysis were finished by Macrogen in Seoul, South Korea. The cDNA libraries were sequenced with an Illumina HiSeq 2500 (Illumina, San Diego, CA, United States) in pair-end mode. A differentially expressed genes (DEG) analysis was performed with edgeR. The genes were selected by *p*-value < 0.05 and fold-change (FC) > 2. The qRT-PCR was performed as described previously (Lee Y. R. et al., 2020). The *RpoZ* gene, encoding DNA-directed RNA polymerase ω-subunit, used at the endogenous reference gene for normalizing levels of RNA. Relative expression of genes was analyzed using the comparative Ct method.

### Determination of Reactive Oxygen Species

The contents of H_2_O_2_ and peroxidase activities were determined using an Amplex^®^ Red Hydrogen Peroxide/Peroxidase Assay Kit (Molecular Probes, Eugene, OR, United States) as described previously (Lee Y. R. et al., 2020). The sonicated cells were prepared in potassium phosphate buffer (pH 7.5). Fifty microliters of sample was mixed with the reaction reagent containing Amplex Red reagent (10-acetyl-3,7-dihydroxyphenoxazine) and horseradish peroxidase (HRP), and then incubated for 30 min according to the manufacturer’s protocol. For measurement of peroxidase activities, hydrogen peroxide was added instead of horseradish peroxidase. Fluorescence was measured using a SYNERGY H1 microplate reader (BioTek, Winooski, VT, United States) with excitation/emission of 530/590 nm.

Intracellular ROS levels were quantified using the fluorescent dye CM-H_2_DCFDA (Invitrogen, Waltham, MA, United States). Just prior to use, CM-H_2_DCFDA was dissolved in ethanol to make 1 mM stock solution. The cells were washed and prepared in 1 mL of PBS buffer (pH 7.4). The CM-H_2_DCFDA stock solution was added to a final working concentration of 1 μM, and mixtures were incubated for 30 min at 30°C. One hundred microliters of samples was transferred to a 96-well black plate. Fluorescence signals were read at excitation of 495 nm and emission of 527 nm.

### Statistical Analysis

Statistical significance of the measurements was determined using Student *t*-test. The data were expressed as mean ± standard deviation. Asterisks indicate significant differences compared with the control group as statistically (*P < 0.05 and ^**^P < 0.01).

## Results and Discussion

### Comparison of Growth Under Hetero- and Autotrophic Conditions in *Rhodobacter sphaeroides*

Chemoautotrophs are important microorganisms that are able to convert CO_2_ into biofuels and biochemicals (Hu et al., 2019). *R. sphaeroides* has versatile metabolic pathways and can assimilate CO_2_ and produce high-value materials such as PHB and carotenoids. However, studies on autotrophic growth have not been sufficiently performed compared to research on heterotrophic growth in *R. sphaeroides.*

**FIGURE 1:**
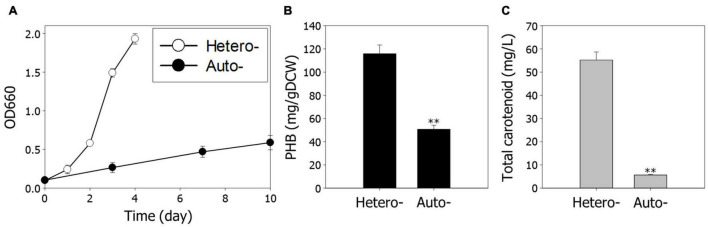
Comparison of cell growth and metabolite production under hetero- and autotrophic conditions in *Rhodobacter sphaeroides*. **(A)** Cell growth under hetero- and autotrophic conditions. The cells were cultivated with different carbon sources, succinic acid, and CO_2_, respectively. **(B)** Contents of PHB. **(C)** Concentrations of total carotenoids. Experiments were conducted in triplicate and error bars indicate standard deviation of mean. Asterisk represents statistically significant difference, as determined by a Student *t*-test (^**^P < 0.01).

To analyze growth characteristics according to different growth conditions, we first examined the growth rate and production of PHB and carotenoids, under hetero- and autotrophic conditions. The overall cell growth is much slower under autotrophic conditions than heterotrophic conditions (Figure 1A). The production of PHB and carotenoids under autotrophic conditions was reduced 2.3-fold and 9.8-fold compared to heterotrophic conditions, respectively (Figures 1B,C). These results indicate that decreased cell growth is a critical impediment for high-value chemical production under autotrophic conditions.

To understand molecular genetic changes that cause growth differences according to culture conditions, we conducted a transcriptomic comparison analysis between hetero- and autotrophic conditions (Table 1). We found that the CBB cycle genes (*cbbLS*, *cfxA*, *prkA*, *cbbR*) for CO_2_ assimilation and the hydrogenase genes (*hupSLDH*, *hypACD*) for uptake H_2_ as an electron source were upregulated in autotrophic conditions. In contrast, the genes involved in the tricarboxylic acid (TCA) cycle (*sucCD*, *mdh*, *sdhAB*, *pykA*, *gltA*, *icd*) for succinic acid consumption were downregulated in autotrophic conditions (Ghirardi et al., 2007; Petushkova et al., 2019; Lee S. Y. et al., 2020). Interestingly, the expression of ROS-related genes, including ROS-scavenging enzymes (*katE*, *katC*, *sodC*, *gpx*), ROS signaling factor (*rpoH*_*II*_, *oxyR*), and redoxins (*trxA*, *grxC*), was elevated in autotrophic conditions compared to heterotrophic conditions (Ziegelhoffer and Donohue, 2009). In bacteria, OxyR, one of ROS signaling transcription factors, plays an important role under oxidative stress. The OxyR regulon has been mainly studied in *E. coli* and regulates the genes which related to elimination of oxidant, maintenance of the balance between thiol groups, and limiting Fe^2+^ availability to minimize the occurrence of the Fenton reaction. When the endogenous levels of uncombined iron are high, resulting in hydroxyl radicals generated by Fenton reaction. OxyR protein is activated the Fur protein, the ferric uptake regulator, resulting in induction of Fe^2+^ binding and iron storage. This regulatory system maintains iron homeostasis and resists to oxidative stress (Andrews et al., 2003; Remes et al., 2014). When the *oxyR* gene was overexpressed, the bacterial cell death was decreased and the specific activities of catalase and superoxide dismutase were increased. When the *oxyR* gene was deleted, the sensitivity to ROS and protein damage was increased (Sun et al., 2002; Ziegelhoffer and Donohue, 2009; Remes et al., 2014). These observations is suggesting that OxyR increases the expression of antioxidant enzymes and protects the cells from ROS. Consistent with previous studies, our observations show that the gene expression of OxyR increased under autotrophic conditions and the gene expression of antioxidants enzymes and thiol groups also increased. It is speculated that OxyR-mediated signaling is important for coping with ROS-induced oxidative stress in *R. sphaeroides*. Altogether, these results support that the transcriptomes involved in various signaling and metabolism were significantly changed between hetero- and autotrophic conditions.

**TABLE 1:** Comparison of transcript expression levels in autotrophic conditions vs. heterotrophic conditions.

Gene number	Gene name	Function	Description	Log_2_(FC)
RSP_1281	*cbbS*	Ribulose 1,5-bisphosphate carboxylase small subunit	Carbohydrate transport and metabolism	3.3
RSP_1282	*cbbL*	Ribulose 1,5-bisphosphate carboxylase large subunit	Energy production and conversion	3.4
RSP_1283	*cfxA*	Fructose-1,6-bisphosphate aldolase	Carbohydrate transport and metabolism	5.0
RSP_1284	*prkA*	Phosphoribulokinase	Energy production and conversion	5.9
RSP_1286	*cbbR*	RuBisCO operon transcriptional regulator, CbbR	Transcription	1.8
RSP_0495	*hupS*	Hydrogenase protein small subunit	Energy production and conversion	10.1
RSP_0496	*hupL*	Hydrogenase protein large subunit	Energy production and conversion	11.2
RSP_0499	*hupD*	Hydrogenase 1 maturation peptidase HyaD	Energy production and conversion	8.6
RSP_0502	*hupH*	HupH hydrogenase expression/formation protein	Posttranslational modification, protein turnover, chaperones	6.7
RSP_0505	*hypA*	Hydrogenase maturation factor HypA	Posttranslational modification, protein turnover, chaperones	5.9
RSP_0508	*hypC*	Hydrogenase maturation protein HypC	Posttranslational modification, protein turnover, chaperones	5.2
RSP_0509	*hypD*	Hydrogenase maturation factor	Posttranslational modification, protein turnover, chaperones	5.0
RSP_0966	*sucD*	Succinyl-CoA synthetase alpha subunit	Energy production and conversion	–2.0
RSP_0967	*sucC*	Succinyl-CoA synthetase (ADP-forming) beta subunit	Energy production and conversion	–2.7
RSP_0968	*mdh*	Malate dehydrogenase	Energy production and conversion	–2.7
RSP_0976	*sdhA*	Succinate dehydrogenase subunit A	Energy production and conversion	–1.9
RSP_0979	*sdhB*	Succinate dehydrogenase catalytic subunit	Energy production and conversion	–1.4
RSP_1766	*pykA*	Pyruvate kinase	Carbohydrate transport and metabolism	–1.6
RSP_1994	*gltA*	Citrate synthase	Energy production and conversion	–1.2
RSP_1559	*icd*	Isocitrate dehydrogenase	Energy production and conversion	–2.4
RSP_2779	*katE*	Catalase	Inorganic ion transport and metabolism	2.8
RSP_2380	*katC*	Catalase	Inorganic ion transport and metabolism	2.5
RSP_1796	*sodC*	Superoxide dismutase	Inorganic ion transport and metabolism	2.4
RSP_2389	*gpx*	Glutathione peroxidase	Posttranslational modification, protein turnover, chaperones	1.3
RSP_0601	*rpoH*_*II*_	RNA polymerase, sigma 32 subunit, RpoH	Transcription	3.2
RSP_0794	*oxyR*	Hydrogen peroxide-inducible genes activator	Transcription	1.8
RSP_1529	*trxA*	Thioredoxin	Posttranslational modification, protein turnover, chaperones	1.1
RSP_1194	*grxC*	Glutaredoxin	Posttranslational modification, protein turnover, chaperones	1.3

**FIGURE 2:**
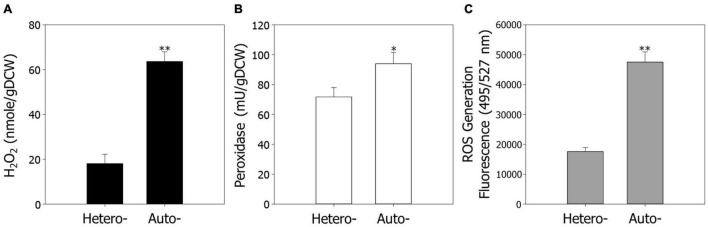
The endogenous levels of ROS under hetero- and autotrophic conditions in *Rhodobacter sphaeroides*. **(A)** Measurement of endogenous hydrogen peroxide. **(B)** The activity of endogenous peroxidase. **(C)** Generation of ROS in cells. ROS were detected using the fluorescence dye CM-H_2_DCFDA and represented in arbitrary units. The fluorescence intensity was normalized to the optical densities of the samples. Experiments were conducted in triplicate and error bars indicate standard deviation of mean. Asterisk represents statistically significant difference, as determined by a Student *t*-test (*P < 0.05 and ^**^P < 0.01).

Previous observations have shown that ROS are closely related to cellular activities, such as cell growth and metabolism (D’Autréaux and Toledano, 2007; de Castro et al., 2013). Furthermore, our data indicate that ROS regulation and signaling play pivotal roles in cell growth under autotrophic conditions. To confirm the results of the transcriptome analysis, we measured the endogenous contents of H_2_O_2_ under hetero- and autotrophic conditions in *R. sphaeroides* (Figure 2A). As predicted, the contents of H_2_O_2_ were 3.5-fold higher under autotrophic conditions than heterotrophic conditions, suggesting that ROS may be one of the factors inhibiting cell growth under autotrophic culture.

We next assayed the activities of peroxidase, which scavenges H_2_O_2_, and endogenous ROS levels under hetero- and autotrophic conditions (Figures 2B,C). Consistent with the transcriptomic analysis, the peroxidase activities were slightly higher under autotrophic conditions, possibly because of induction of ROS as a substrate. The fluorescence of ROS generation levels, which were measured through a ROS indicator, CM-H_2_DCFDA, were 2.7-fold higher under autotrophic growth conditions.

It was unexpected that ROS levels would be higher under autotrophic conditions, including only CO_2_, H_2_, and Ar, because ROS are generally produced in the presence of oxygen. This indicates that autotrophic metabolism may causes cellular changes and oxidative stress by generating ROS. There have been various reports on the association between CO_2_ and ROS in plants, which have been heavily studied with regard to CO_2_ assimilation. High concentrations of CO_2_ mediates important signaling in stomatal movement induced by an increase of ROS in plants (Ma and Bai, 2021). It was also reported that 10% of CO_2_ promoted the activities and expression of antioxidant enzymes such as catalase (CAT), glutathione peroxidase (GPX), and superoxide dismutase (SOD) in pears (Wang et al., 2021), similar to our experimental results. ROS are a critical inhibitory factor that profoundly affect CO_2_ fixation. Intracellular ROS potently inhibit CO_2_ fixation by interacting with either Calvin-Benson cycle enzymes or intermediates and reducing their expression (Sharma et al., 2012). Underlying molecular mechanisms and association between CO_2_ and ROS in chemoautotrophs such as *R. sphaeroides* are still largely unknown. We therefore propose that the effects of ROS on cell growth and metabolism under autotrophic conditions should be further investigated in diverse organisms.

### Isolation and Characterization of YR-1 Mutant

Cell growth and production of useful metabolites were significantly reduced in autotrophic conditions compared to heterotrophic conditions. In order to increase the cell growth rate and metabolite productivity, it is necessary to innovatively improve the performance of strain. To achieve this, we carried out random mutagenesis using *N*-methyl-*N*’-nitro-*N*-nitrosoguanidine (NTG), which can cause various mutations in the genome, on the wild-type *R. sphaeroides* strain. To establish the concentration of CO_2_ in experimental conditions, we investigated cell growth and the endogenous ROS according to different CO_2_ concentration (Supplementary Figure 1). Although the endogenous levels of ROS were slightly higher at 10% of CO_2_ than at 5% of CO_2_, cell growth did not differ significantly. General CO_2_ concentrations in flue gases are around 10% (Wang and Song, 2020). Considering that flue gases are directly used without separation and purification processes, we conducted under 10% of CO_2_ conditions.

**FIGURE 3:**
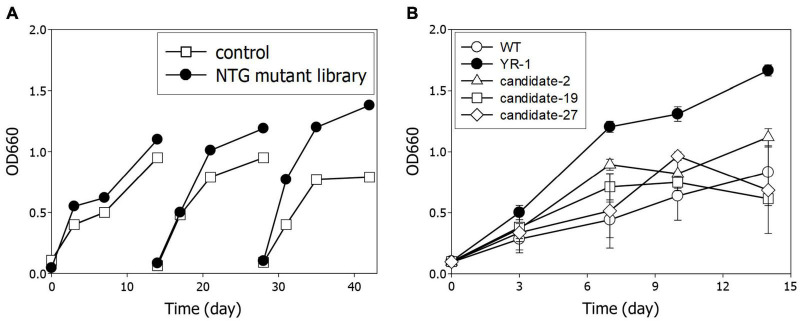
Isolation of YR-1 mutant. **(A)** Serial transfer of the NTG mutant library. Experiments were subcultured three times and cultivated under 10% of CO_2_ and 60% of H_2_. **(B)** Screening of mutant for isolation of YR-1. The candidates were randomly selected and growth was estimated under 10% of CO_2_ and 60% of H_2_. Experiments were conducted in triplicate and error bars indicate standard deviation of mean.

Next, we performed serial transfer culture under autotrophic conditions with 10% of CO_2_ and 60% of H_2_ to enrich the dominant mutants with accelerated cell growth. In the third round, it was observed that the growth of the NTG mutant library was meaningfully increased relative to the wild-type strain (Figure 3A). To isolate a predominant single colony, we randomly selected more than 50 colonies and assayed cell growth and CO_2_ consumption of each candidate. Among them, the YR-1 mutant showed the highest growth rate, which was about two times faster than the wild-type strain (Figure 3B). Microalgae are typically useful candidates for CO_2_ fixation due to their rapid growth rate and high photosynthetic efficiency (Ma et al., 2022). According to our results, the YR-1 mutant shows a growth rate similar to that of some microalgae, such as *Chlorella vulgaris*, *Nannochloropsis oculata*, under autotrophic conditions supplied with 10% of CO_2_ (Chiu et al., 2009; Lakshmikandan et al., 2020). *R. sphaeroides* has the advantage of the production of various product due to versatile metabolism and well-establish genetic engineering, as well as simplicity of cell lysis and harvest of metabolites. If a growth rate and productivity of valuable metabolite is more improved through genetic manipulation and optimization of culture conditions, *R. sphaeroides* can also be good CO_2_ converting microorganisms.

Although NTG is a very powerful mutagen, it is difficult to predict the exact mutation site. To characterize the YR-1 mutant, we performed a genome sequencing analysis. Compared with the wild-type genome, we found 36 variants, including 20 non-synonymous and 16 synonymous variants, in the YR-1 genome. We reported annotations of frameshift variants and a stop gain variant in Table 2. The annotation of all variants in YR-1 mutant except to frameshift variants and a stop gain variant is reported in Supplementary Table 1. The frameshift variants occurred in three positions, the ubiquinone biosynthetic gene, PHB biosynthetic gene, and an uncharacterized gene. The stop gain variant was found in a gene of the ABC efflux transporter, which has ATPase activity.

**TABLE 2:** Variant annotation in YR-1.

Chromosome	Position	Reference bases	Alternate bases	START	END	Description	Name
1	468375	.	G	467543	468439	Four iron, four sulfur cluster binding, metal ion binding, peptidase activity, ubiquinone biosynthetic process	*ubiV*, Ubiquinone biosynthesis protein UbiV
1	554499	.	A	552855	554660	Transferase activity, transferring acyl groups, polyhydroxybutyrate biosynthetic process	Poly-beta-hydroxybutyrate polymerase
1	2017824	.	T	2016566	2017858		Uncharacterized protein
1	1797222	G	A	1795441	1797228	ATPase activity, ATPase-coupled transmembrane transporter activity, ATP binding	ABC efflux transporter, fused ATPase and inner membrane subunits

Ubiquinone is involved in respiratory electron transport chain in bacteria. It is also well-known that ubiquinol, a reduced from of ubiquinone, acts as an antioxidant that efficiently removes free radicals (Ernster and Forsmark-Andrée, 1993). It has been reported that the UbiV protein is involved in O_2_-independent hydroxylation by forming a heterodimer with UbiU, unlike the general ubiquinone biosynthesis (Pelosi et al., 2019). Although the open reading frame of the *ubiV* gene was disturbed in the YR-1 mutant genome, it occurred at the end of the gene, resulting in the length of the total UbiV protein being longer by 14 amino acids. To evaluate the effect caused by disruption of *ubiV* gene, we also analyzed the content of ubiquinone in wild-type and YR-1 mutant (Supplementary Figure 2A). The result of analyze was confirmed that the biosynthesis of ubiquinone is 4.9-fold decreased in the mutant compared to the wild-type. This suggests that changes in the ubiquinone biosynthesis and antioxidant activity may occur in *R. sphaeroides*. A previous work showed that PHB production is closely related to ROS generation (Lee Y. R. et al., 2020). Since the *phaC* gene, a key PHB biosynthesis enzyme gene, was disrupted in the YR-1 mutant genome, it is expected that it affects PHB and ROS production. The uncharacterized protein, encoded by RSP_3764 locus, also has a frameshift variant. It occurred at the end of gene, resulting in the length of the total protein being shorter by 74 amino acids. The results of protein sequence analysis using SMART are represent that this protein has two RPT1 domain, as known to the internal repeat domain (Supplementary Figure 2B). This domain is involved in protein-protein interaction with various cellular proteins and regulated the transcriptional activity (Das et al., 2009). This protein has the potential, however, the function of proteins is not clearly. It is necessary to the further research on the function of unknown protein and the interaction of another proteins. The RSP_2254 locus, which has a mutation that stops gene synthesis, has been identified as an ABC transporter. In general, the ABC transporter functions are important in response to oxidative stress (Ohtsu et al., 2015; Grewal et al., 2017). In addition, the missense variant was also found in the genes related to biosynthesis of bacteriochlorophyll, cytochrome *c* oxidase, and some of ABC transporter. The *coxI* gene, which encoded cytochrome *c* oxidase, changed from T to C in 728th base pair, resulting that the 234rd amino acid changed from leucine to proline. This protein, encoding by *coxI*, may have been changed in protein structure. Because the changes in amino acid sequence to proline is known to influence in protein structure. Based on both previous and our own data, we posit that synergetic effect caused by various mutations in the YR-1 mutant genome sequence have a profound effect on ROS regulation, enhancing cell growth and CO_2_ consumption efficiency under autotrophic conditions. For further research, it is necessary to clarify which gene is the key factors of phenotype in YR-1 mutant by generating a single knockout mutant strain.

### Improvement of Growth and Cell Viability by Reactive Oxygen Species Regulation

To elucidate the cause of the accelerated growth of YR-1 mutant, we performed a transcriptomic analysis. By comparing the expression levels between wild-type and YR-1 mutant, we obtained up-regulated genes in YR-1 mutant after screening for >2-fold changes and with *p* < 0.05 (Table 3). The results of the analysis revealed that the transcript levels of *cbbLS*, encoding the ribulose 1,5-bisphosphate carboxylase involved in CO_2_ fixation, and the levels of *hupLDH* and *hypAD*, encoding the hydrogenase for hydrogen uptake, were more than 2-fold higher than in the YR-1 mutant. Also, the expression of genes associated with the TCA cycle (*pdhAa*, *pdhAb*, *sdhB*, *icd*, *frdB*) was elevated. Upregulating the expression of enzymes involved in CO_2_ assimilation improved the efficiency of CO_2_-fixation, promoting overall metabolism (Behler et al., 2018). Additional supplementation of NADH through the expression of heterologous hydrogenase increased hydrogen production in *E. coli* (Lamont and Sargent, 2017). It was also reported that enhancement of energy production *via* increased transcript levels of the TCA cycle leads to an increase in microalgal biomass (Paik et al., 2019). These findings suggest that the changes of expression of genes involved in carbon assimilation and energy conversion affected the enhancement of growth in YR-1 under autotrophic conditions.

**TABLE 3:** Comparison of transcript expression levels in YR-1 vs. wild-type.

Gene number	Gene name	Function	Description	Log_2_(FC)
RSP_1281	*cbbS*	Ribulose 1,5-bisphosphate carboxylase large subunit	Carbohydrate transport and metabolism	1.6
RSP_1282	*cbbL*	Ribulose 1,5-bisphosphate carboxylase small subunit	Energy production and conversion	1.6
RSP_0496	*hupL*	Hydrogenase protein large subunit	Energy production and conversion	1.2
RSP_0499	*hupD*	Hydrogenase 1 maturation peptidase HyaD	Energy production and conversion	2.4
RSP_0502	*hupH*	HupH hydrogenase expression/formation protein	Posttranslational modification, protein turnover, chaperones	2.5
RSP_0505	*hypA*	Hydrogenase maturation factor HypA	Posttranslational modification, protein turnover, chaperones	1.8
RSP_0509	*hypD*	Hydrogenase maturation factor	Posttranslational modification, protein turnover, chaperones	1.3
RSP_4047	*pdhAa*	Pyruvate dehydrogenase E1 component subunit alpha	Energy production and conversion	1.5
RSP_4049	*pdhAb*	Pyruvate dehydrogenase E1 component subunit beta	Energy production and conversion	1.4
RSP_0979	*sdhB*	Succinate dehydrogenase catalytic subunit	Energy production and conversion	1.0
RSP_1559	*icd*	Isocitrate dehydrogenase	Energy production and conversion	1.2
RSP_3150	*frdB*	Succinate dehydrogenase iron-sulfur subunit	Energy production and conversion	1.1
RSP_1826	*coxII*	Cytochrome *c* oxidase subunit 2	Energy production and conversion	1.9
RSP_1828	*ctaG*	Cytochrome *c* oxidase assembly protein CtaG	Posttranslational modification, protein turnover, chaperones	1.1
RSP_1829	*coxIII*	Cytochrome *aa*_3_ subunit 3	Energy production and conversion	1.4
RSP_1877	*coxI*	Cytochrome *c* oxidase subunit 1	Energy production and conversion	1.8
RSP_2785	*cycF*	Cytochrome *c*-554	Energy production and conversion	1.5
RSP_2512	*nuoA*	NADH-quinone oxidoreductase subunit A	Energy production and conversion	2.0
RSP_2513	*nuoB1*	NADH-quinone oxidoreductase subunit B1	Energy production and conversion	1.6
RSP_2514	*nuoC*	NADH-quinone oxidoreductase subunit C	Energy production and conversion	1.7
RSP_2515	*nuoD*	NADH-quinone oxidoreductase subunit D	Energy production and conversion	2.0
RSP_2516	*nuoE*	NADH dehydrogenase subunit E	Energy production and conversion	1.1
RSP_2518	*nuoF*	NADH-quinone oxidoreductase subunit F	Energy production and conversion	1.1
RSP_2779	*katE*	Catalase	Inorganic ion transport and metabolism	2.2
RSP_2380	*katC*	Catalase	Inorganic ion transport and metabolism	2.2
RSP_1796	*sodC*	Superoxide dismutase	Inorganic ion transport and metabolism	1.4
RSP_2389	*gpx*	Glutathione peroxidase	Posttranslational modification, protein turnover, chaperones	1.6
RSP_1092	*rpoE*	ECF RNA polymerase sigma factor RpoE	Transcription	2.5
RSP_1093	*chrR*	Anti-sigma-E factor ChrR	Transcription	1.6
RSP_2143	*phrA*	DNA photolyase, Cryptochrome 1 apoprotein (Blue light photoreceptor)	Replication, recombination, and repair	1.2
RSP_0601	*rpoH*_*II*_	RNA polymerase, sigma 32 subunit, RpoH	Transcription	3.3
RSP_1529	*trxA*	Thioredoxin	Posttranslational modification, protein turnover, chaperones	1.0
RSP_3127	*arsC*	Arsenate reductase (glutaredoxin)	Inorganic ion transport and metabolism	4.9

Energy generation is sensitively regulated by external growth conditions such as aerobic/anaerobic respiration and anaerobic photosynthesis, and has a significant impact on cell growth in *R. sphaeroides* (Pappas et al., 2004). Expression of the genes involved in components of the electron transport chain, such as cytochrome *c* oxidase of the *aa*_3_ type (*coxI*, *coxII*, *coxIII*, *ctaG*, *cycF*) and NADH-quinone oxidoreductase (*nuoABCDEF*), was induced in YR-1 mutant (Flory and Donohue, 1997; Mouncey and Kaplan, 1998). Although these genes are known to be upregulated in the presence of oxygen, their expression also increased under anaerobic conditions in our mutant, supposing that numerous genetic variations through NTG may have effected. Recently, it was reported that terminal oxidases of the bacterial respiratory chain serve as a defense system against ROS (Borisov et al., 2021). Together, the upregulation of genes involved in the electron transport chain may be meaningful with regard to ROS regulation in YR-1 mutant.

Furthermore, the expression of ROS-related genes, including ROS-scavenging enzyme (*katE*, *katC*, *sodC*, *gpx*), ROS signaling factor (*rpoE*, *chrR*, *phrA*, *rpoH*_*II*_), and redoxins (*trxA*, *arsC*), was also increased in YR-1 mutant. Interestingly, the expression of genes encoding the singlet oxygen stress response regulon, σ*^E^*–ChrR regulon, is upregulated in the mutant. The σ*^E^*–ChrR regulon includes the regulator (RpoE), its inhibitor (ChrR), and the several proteins involved in the cellular response to singlet oxygen, including DNA photolyase; cryptochrome 1 apoprotein and RNA polymerase RpoH_*II*_. This regulon responds to singlet oxygen, resulting in protection of cells from oxidative stress and repair of damage caused by ROS (Anthony et al., 2005; Dufour et al., 2008; Ziegelhoffer and Donohue, 2009). *R. sphaeroides* is a facultative microorganisms, which has the ability of growth using bacterial photosynthesis under autotrophic conditions. The formation of ^1^O_2_ is unavoidable during the utilization of light energy by bacterial photosynthesis. In previous studies, the activity of RpoE was significantly enhanced when it was response to singlet oxygen stress. In RpoE-deficient cells, the singlet oxygen was rapidly produced and caused the cell death (Anthony et al., 2005). These results are suggesting that the regulation of singlet oxygen generated during photosynthesis by σ*^E^*–ChrR regulon is important to maintain cell viability. The DNA photolyase, encoded by *phrA*, is repaired the light-induced damage in DNA. RpoH_*II*_, the alternative sigma-factor of the heat shock family, is directly regulated by RpoE and also activated by ^1^O_2_. Glutathione peroxidase, encoded by RSP_2389, is one of the genes that the translation directly regulated by RpoH_*II*_. This enzyme is known to be a key enzyme of defense ROS (Ursini et al., 1995; Mittler et al., 2004; Green and Donohue, 2006; Hendrischk et al., 2007). The various transcriptional response in ROS-signaling and ROS-scavenging enzyme is modulated by σ*^E^*–ChrR regulon. It is supported that YR-1 mutant may have more resistance to singlet oxygen by activation of this regulon. It has also been reported that transcriptional responses to ROS determine tolerance to ROS in *S. cerevisiae* (Gulshan et al., 2011). Consequently, we suggest that the upregulation of genes involved in ROS scavenging and signaling is strongly associated with enhancement of growth in YR-1 under autotrophic conditions.

**FIGURE 4:**
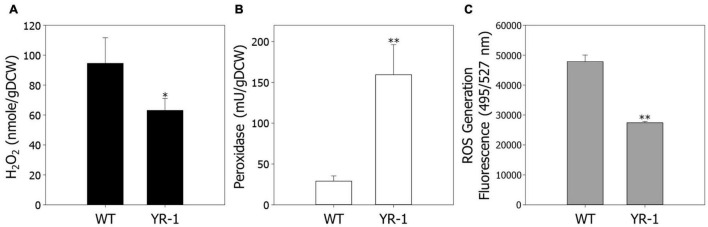
Comparison of endogenous ROS levels in the wild-type and YR-1 mutant. Precultured cells were inoculated into 100 mL of modified Sistrom’s medium in serum bottles. **(A)** Measurement of endogenous hydrogen peroxide. **(B)** The activity of endogenous peroxidase. **(C)** Generation of ROS in cells. ROS were detected using the fluorescence dye CM-H_2_DCFDA and represented in arbitrary units. The fluorescence intensity was normalized to the optical densities of the samples. Experiments were conducted in triplicate and error bars indicate standard deviation of mean. Asterisk represents statistically significant difference, as determined by a Student *t*-test (*P < 0.05 and ^**^P < 0.01).

Regulation of ROS has a crucial role in cell viability and metabolic processes under autotrophic conditions. To confirm ROS regulation based on the transcriptome analysis, we next examined the levels of H_2_O_2_ in wild-type and YR-1 mutant (Figure 4A). To secure additional bacterial cell for various analysis, the experiments were conducted in 100 mL of working volume. The levels of H_2_O_2_ were approximately 33% decreased in YR-1 compared to the wild-type. We subsequently measured the activities of peroxidase and endogenous ROS contents in wild-type and YR-1 (Figures 4B,C). Compared with the wild-type, the peroxidase activities were 5.5-fold higher and the contents of endogenous ROS were 42% lower in YR-1 mutant. These results indicate that controlling ROS in cells is important to accelerate autotrophic growth. It has been reported that peroxidase activity is sensitively affected by culture conditions such as aeration and supplementation and it is presumed that peroxidase activity of wild type slightly changed as the experimental working volume increased (Falade et al., 2020). Many studies seeking to explain the correlation between regulation of ROS and cell viability have been reported. Managing the homeostasis of ROS through oxidant scavenging and ROS signaling mitigates the toxicity of ROS and increases the cell viability (D’Autréaux and Toledano, 2007). The activities of ROS-scavenging enzymes, such as catalase (CAT) and superoxide dismutase (SOD), are delicately regulated during exponential and stationary growth phases in *Phycomyces blakesleeanus* (de Castro et al., 2013). Moreover, post-oxidative stress caused by ROS is known to mediate cell death in bacteria. This can be partially overcome by the introduction of an exogenous mitigating agent that prevent the accumulation of ROS (Hong et al., 2019). The modulation of ROS is critical to maintain viability in various organisms. Our results also support that ROS regulation helps to improve cell growth under autotrophic conditions in *R. sphaeroides*. Further analysis of antioxidants, including chemicals and enzymes, will provide additional clues as to how ROS modulate cell growth.

### Enhancement of Carotenoid Production in YR-1 Mutant

**FIGURE 5:**
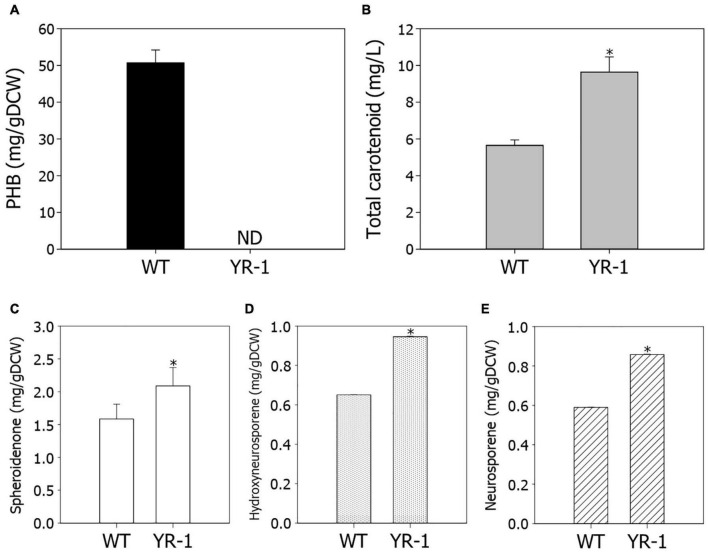
Measurement of metabolite production in the wild-type and YR-1 mutant. **(A)** Contents of PHB. **(B)** Concentrations of total carotenoids. **(C)** Contents of spheroidenone. **(D)** Contents of hydroxyneurosporene. **(E)** Contents of neurosporene. Analyses were performed in triplicate and error bars indicate standard deviation of mean. Asterisk represents statistically significant difference, as determined by a Student *t*-test (*P < 0.05).

The conversion of CO_2_ into valuable chemicals is the crucial step for biorefinery of CO_2_. We investigated the potential for production of high value-added chemicals in YR-1 mutant, which grew faster under autotrophic conditions. PHB is a type of biopolymer produced by microorganisms, which is regarded as a promising alternative for polypropylene. Due to its biodegradability and biocompatibility, it has become commercially attractive (Eroglu et al., 2010). In *R. sphaeroides*, PHB is the most widely known metabolite as a carbon storage compound; however, it was not detected in YR-1 mutant (Figure 5A). It is considered that PHB does not accumulate and various metabolisms derived from acetyl-CoA are altered because the *PhaC* gene is disrupted in the YR-1 mutant. Inactivation of the PHB biosynthetic pathway leads to improved cell growth and production of other high-value materials, such as isoprenoid and hydrogen (Kim et al., 2006; Orsi et al., 2020). These results suggest that the production of metabolites other than PHB was increased by disruption of the *PhaC* gene under autotrophic conditions in YR-1 mutant. Furthermore, it is needed to investigate where the rest carbon flux, which was caused by disruption of PHB biosynthesis, was directed.

*Rhodobacter* is a strain with high pigment accumulation *via* a well-developed carotenoid biosynthetic pathway. We examined the production of carotenoids in YR-1 mutant. The production of total carotenoids were approximately 1.7-fold higher in YR-1 mutant compared to the wild-type through enhancement of cell growth (Figure 5B). Unfortunately, the RNA-sequencing results did not reliably confirm the expression of carotenoid biosynthetic genes. To compare transcript levels of carotenoid biosynthetic genes between wild-type and YR-1 mutant, we additionally performed the quantitative real-time PCR (qRT-PCR) (Supplementary Figure 3). The relative expression of carotenoid biosynthetic genes, such as *crtA*, *crtB*, *crtC*, *crtD*, *crtF*, *crtI*, were slightly higher in YR-1 mutant compared to wild-type. Because the gene expression showed slight difference, we next measured the specific carotenoid in *R. sphaeroides*, which synthesized by genes of carotenoid biosynthesis. Except for spheroidene, the contents of spheroidenone, hydroxyneurosporene, and neurosporene, which are known as representative carotenoids in *R. sphaeroides*, were slightly higher in YR-1 mutant than in the wild-type (Figures 5C–E and Supplementary Figure 4). These results indicate that carotenoid biosynthesis was promoted in the YR-1 mutant. The pathway of carotenoid and PHB biosynthesis *via* CBB pathway represent in Supplementary Figure 5. Based on our results, we suspected that the disruption of *phaC* gene through NTG mutation caused inactivation of PHB biosynthesis, resulting that the remaining carbon flux may have shifted into biosynthesis of carotenoids in YR-1 mutant. Although further research is also needed to clarify the cause and to confirm our hypothesis, our results are suggesting that the various mutations by NTG promote biosynthesis of specific carotenoid under autotrophic conditions.

Carotenoids are high value-added compounds that are widely used in various industrial applications. Whereas chemically synthesized carotenoids have low activity and unproven safety limitations, biologically synthesized carotenoids are considered a useful alternative to overcome safety issues and efficiently reduce greenhouse gases (Liu et al., 2021). In particular, it is possible to produce carotenoids by directly fixing CO_2_ in autotrophic microorganisms, such as microalgae. Diverse strategies to increase the production yield of astaxanthin, a key carotenoid in microalgae, have been reported. High levels of light and CO_2_ significantly enhanced the accumulation of astaxanthin in *Haematococcus pluvialis* (Christian et al., 2018). The *H. pluvialis* mutants induced by nuclear irradiation showed increased biomass and astaxanthin yields through gradient domestication in flue gas containing high concentrations of CO_2_. In addition, the yield was further increased by optimizing the nitrogen and phosphorus concentrations in the medium (Cheng et al., 2016). These previous studies suggest that carotenoid production can be further increased through the optimization of autotrophic growth conditions, such as CO_2_ concentration, light intensity, and medium composition, even in *R. sphaeroides*.

Carotenoids also act as natural antioxidants that protect cells from oxidative stress by quenching singlet oxygen. Carotenoid-deficient mutants of *R. sphaeroides* showed a lower survival rate under photooxidative stress conditions, indicating that carotenoids are important to responses against ROS (Glaeser and Klug, 2005). Spheroidenone, which is abundant in *R. sphaeroides*, has excellent antioxidant activity among carotenoids and greatly contributes to reducing oxidative damage, especially in aerobic conditions (Licht et al., 2020). In our data, the contents of spheroidenone were increased in YR-1 mutant compared with the wild-type. Although the molecular mechanism for increasing the content of spheroidenone has not been fully elucidated yet, our findings suggest that YR-1 mutant may be more resistant to environments with high levels of ROS due to elevated overall carotenoid production.

## Conclusion

Biological CO_2_ utilization has been researched mainly dependent on photosynthesis until now, where light and low growth rates are major obstacles. To overcome the slow growth rate under autotrophic conditions, we carried out isolation of mutants that were treated by NTG in *R. sphaeroides*. The selected a mutant, YR-1, showed increased cell growth and carotenoid production under autotrophic conditions. Furthermore, we found that the levels of ROS were much lower in YR-1 mutant compared to the wild-type. Altogether, our observations suggest that controlling ROS is important to promote cell growth and carotenoid production under autotrophic conditions.

## Data Availability Statement

The authors acknowledge that the data presented in this study must be deposited and made publicly available in an acceptable repository, prior to publication. Frontiers cannot accept a manuscript that does not adhere to our open data policies.

## Author Contributions

SL contributed to conceptualization and design of the study. YL and W-HL performed the experiments and data acquisition. YL, W-HL, and SL wrote the first draft of the manuscript. SYL, JL, M-SK, MM, GP, HK, J-IK, J-SL, and SL provided critical comments and contributed to the discussion of the results followed by writing and reviewing the manuscript. J-SL and SL provided resources and funding. All authors contributed to manuscript revision, read, and approved the submitted version.

## Conflict of Interest

The authors declare that the research was conducted in the absence of any commercial or financial relationships that could be construed as a potential conflict of interest.

## Publisher’s Note

All claims expressed in this article are solely those of the authors and do not necessarily represent those of their affiliated organizations, or those of the publisher, the editors and the reviewers. Any product that may be evaluated in this article, or claim that may be made by its manufacturer, is not guaranteed or endorsed by the publisher.

## Supplementary Material

Supplementary Figure 1 **(A)** Comparison of cell growth under CO_2_ concentration of 5 and 10% in *Rhodobacter sphaeroides*. Precultured cells were inoculated into 20 mL of modified Sistrom’s medium in serum bottles. The cultures were incubated under at 30°C, 150 rpm, and purged with a gas composition of CO_2_ 5%, H_2_ 60%, argon 35% and CO_2_ 10%, H_2_ 60%, argon 30%, respectively. **(B)** The endogenous levels of ROS under CO_2_ concentration of 5 and 10%. ROS was measured using CM-H_2_DCFDA and represented in arbitrary units. The fluorescence intensity was normalized to the optical densities of the samples. Experiments were conducted in triplicate and error bars indicate standard deviation of mean. Asterisk represents statistically significant difference, as determined by s Student *t*-test (**P* < 0.05).

Supplementary Figure 2 **(A)** Contents of ubiquinone in wild-type and YR-1 mutant. Experiments were conducted in triplicate and error bars indicate standard deviation of mean. Asterisk represents statistically significant difference, as determined by s Student *t*-test (^**^*P* < 0.01). **(B)** Protein domain analysis of RSP_3764 locus in YR-1 mutant. RPT1, internal repeat domain.

Supplementary Figure 3 Expression of genes encoding carotenoid biosynthetic enzymes. *crtA*, spheroidene monooxygenase (RSP_0272); *crtB*, phytoene synthase (RSP_0270); *crtC*, hydroxyneurosporene dehydrogenase (RSP_0267); *crtD*, methoxyneurosporene dehydrogenase (RSP_0266); *crtF*, hydroxyneurosporene-O-methyltransferase (RSP_0264); *crtI*, phytoene dehydrogenase (RSP_0271).

Supplementary Figure 4 Contents of spheroidene in wild-type and YR-1 mutant.

Supplementary Figure 5 PHB and carotenoid biosynthesis pathways and genes in *Rhodobacter sphaeroides*.
