# Supplementary figures and images for: Regulation of Reactive Oxygen Species Promotes Growth and Carotenoid Production Under Autotrophic Conditions in Rhodobacter sphaeroides
Source: Front Microbiol. 2022 Feb 28;13:847757. doi: 10.3389/fmicb.2022.847757 (PMC8920488; doi:10.3389/fmicb.2022.847757)

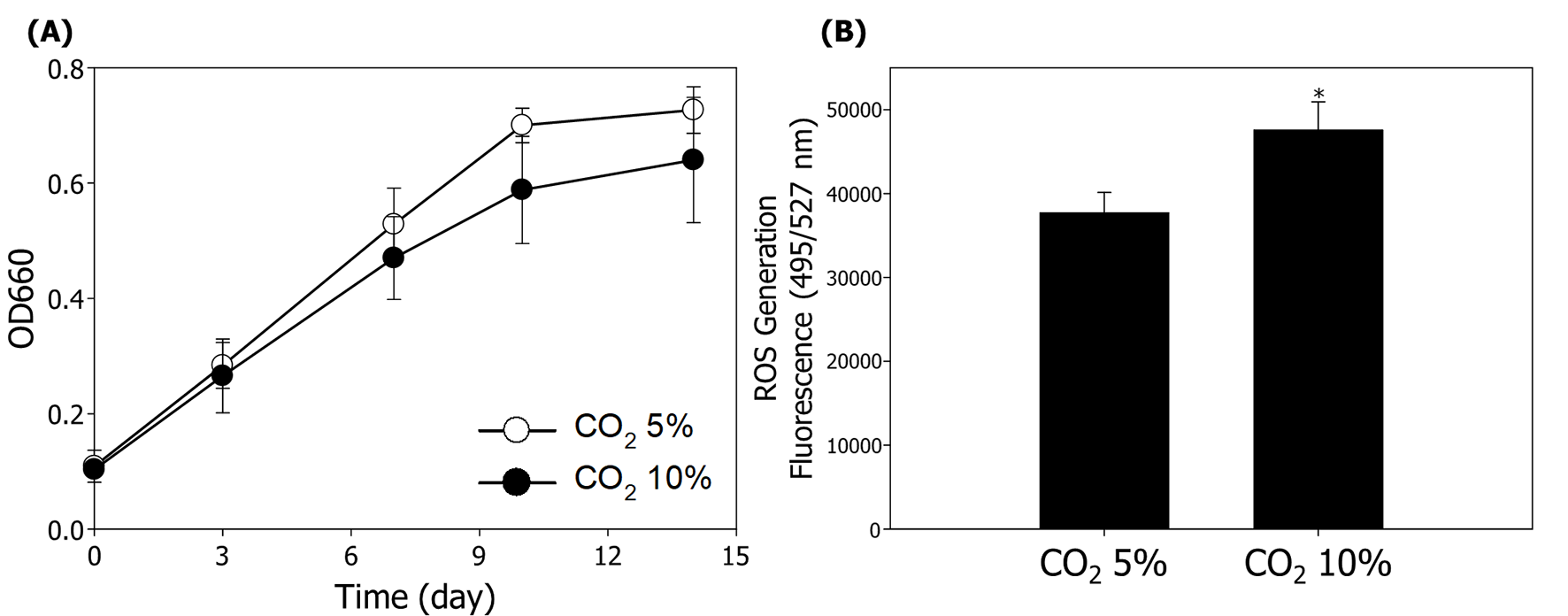

Supplement: Supplementary Figure 1 — (A) Comparison of cell growth under CO2 concentration of 5 and 10% in Rhodobacter sphaeroides. Precultured cells were inoculated into 20 mL of modified Sistrom’s medium in serum bottles. The cultures were incubated under at 30°C, 150 rpm, and purged with a gas composition of CO2 5%, H2 60%, argon 35% and CO2 10%, H2 60%, argon 30%, respectively. (B) The endogenous levels of ROS under CO2 concentration of 5 and 10%. ROS was measured using CM-H2DCFDA and represented in arbitrary units. The fluorescence intensity was normalized to the optical densities of the samples. Experiments were conducted in triplicate and error bars indicate standard deviation of mean. Asterisk represents statistically significant difference, as determined by s Student t-test (*P < 0.05). [file Image_1.TIF]

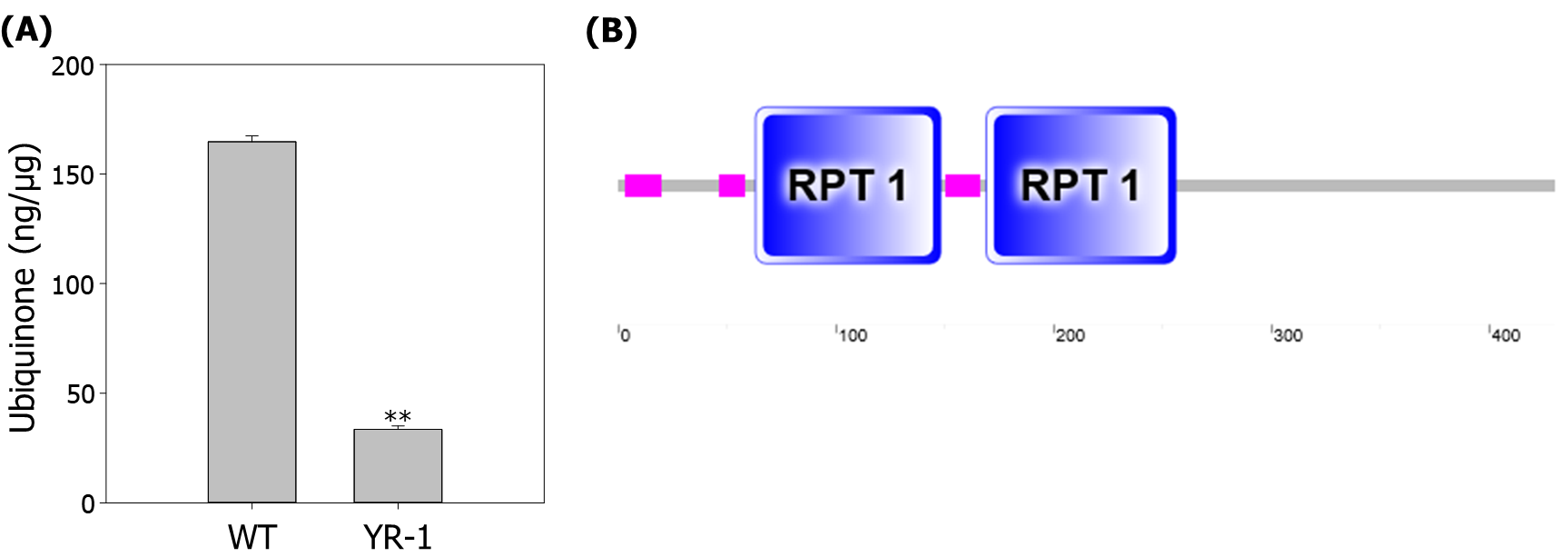

Supplement: Supplementary Figure 2 — (A) Contents of ubiquinone in wild-type and YR-1 mutant. Experiments were conducted in triplicate and error bars indicate standard deviation of mean. Asterisk represents statistically significant difference, as determined by s Student t-test (**P < 0.01). (B) Protein domain analysis of RSP_3764 locus in YR-1 mutant. RPT1, internal repeat domain. [file Image_2.TIF]

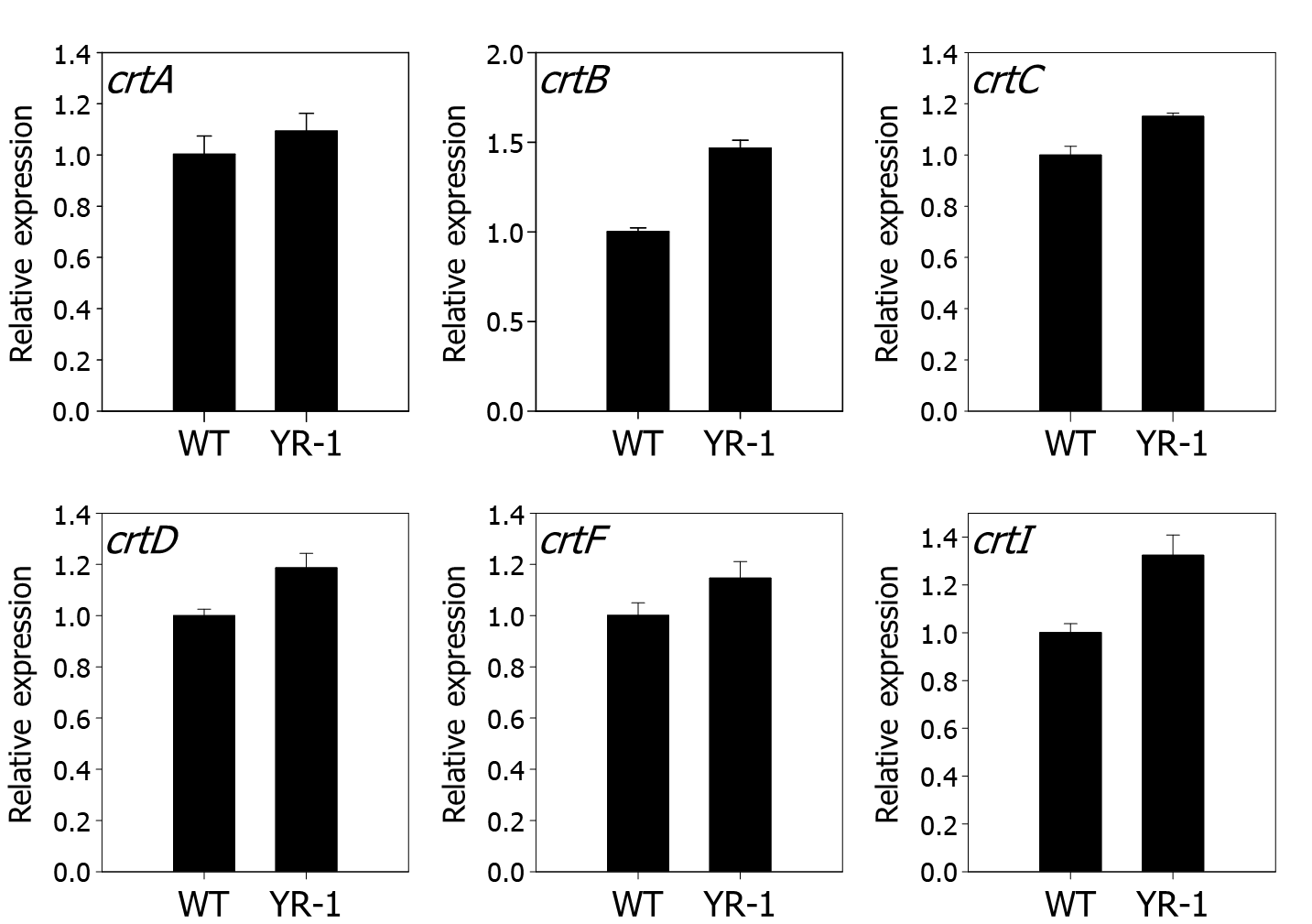

Supplement: Supplementary Figure 3 — Expression of genes encoding carotenoid biosynthetic enzymes. crtA, spheroidene monooxygenase (RSP_0272); crtB, phytoene synthase (RSP_0270); crtC, hydroxyneurosporene dehydrogenase (RSP_0267); crtD, methoxyneurosporene dehydrogenase (RSP_0266); crtF, hydroxyneurosporene-O-methyltransferase (RSP_0264); crtI, phytoene dehydrogenase (RSP_0271). [file Image_3.TIF]

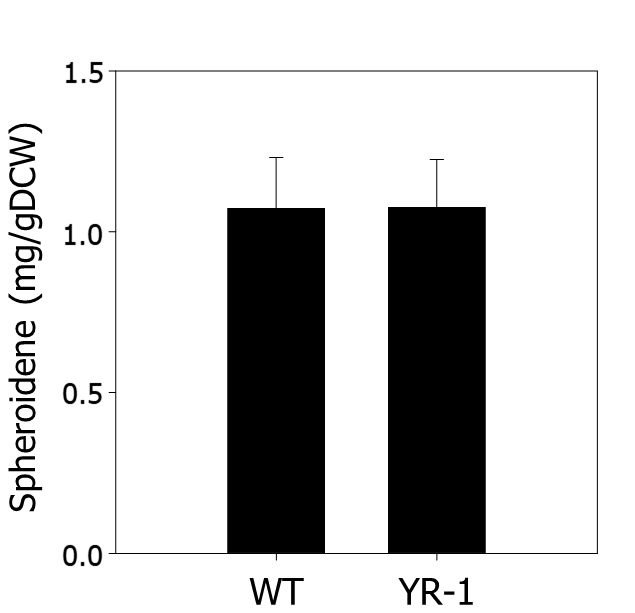

Supplement: Supplementary Figure 4 — Contents of spheroidene in wild-type and YR-1 mutant. [file Image_4.TIF]

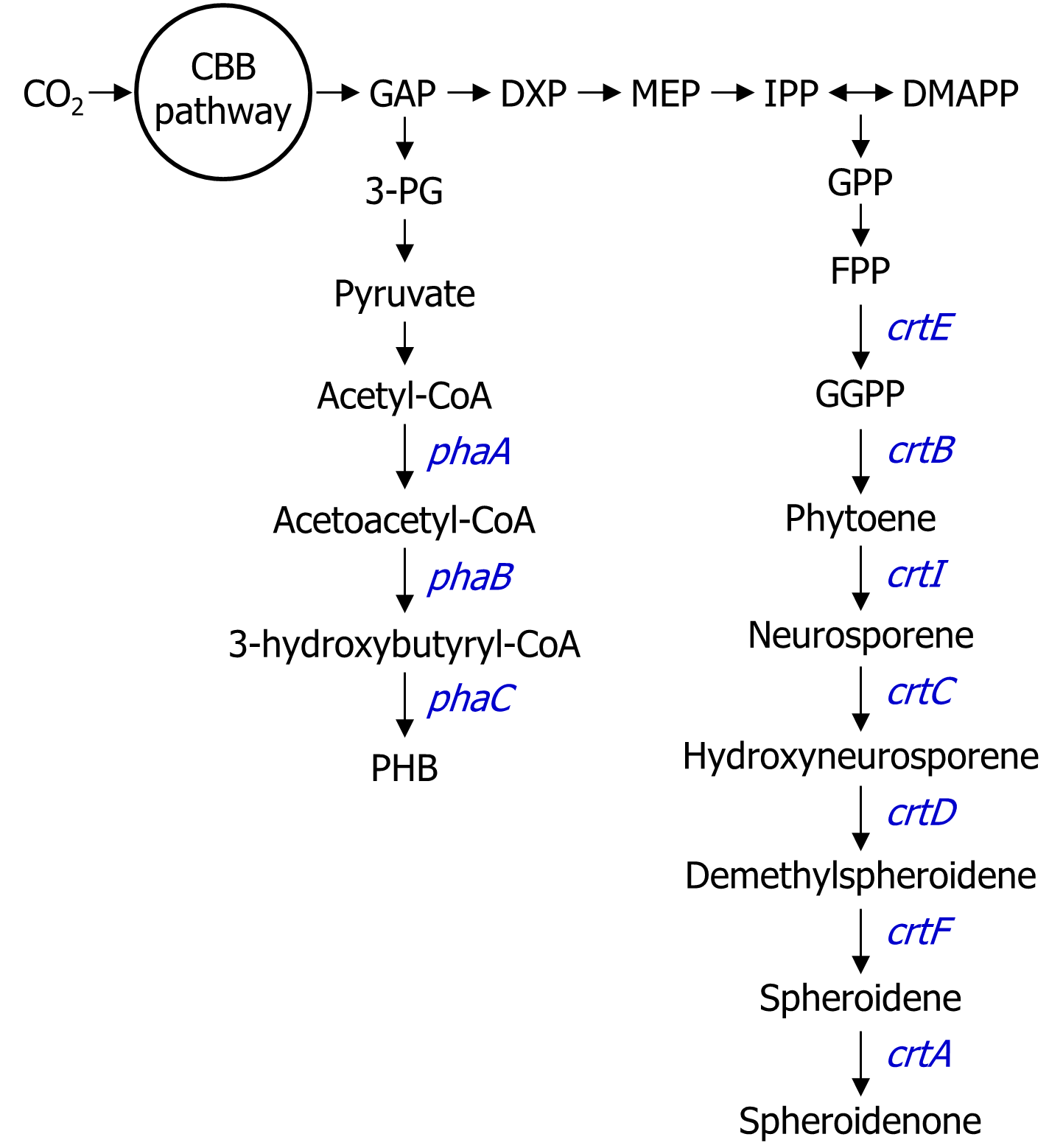

Supplement: Supplementary Figure 5 — PHB and carotenoid biosynthesis pathways and genes in Rhodobacter sphaeroides. [file Image_5.TIF]
